# Effect of Immediate Dentin Sealing on the Bond Strength of Resin Cement to Diabetic Dentin

**DOI:** 10.3390/biomimetics11090671

**Published:** 2026-09-18

**Authors:** Ayşenur Altuğ Yıldırım, Beyza Arslandaş Dinçtürk, Cemile Kedici Alp

**Affiliations:** 1 Çankırı Faculty of Dentistry, Karatekin University, Uluyazı Campus, Çankırı 18100, Türkiye; 2 Faculty of Dentistry, Gazi University, Bişkek Cd. (8. Cd.) 1. Sk. No: 8, Ankara 06490, Türkiye; beyzaarslandas@gazi.edu.tr (B.A.D.); cemilealp@gazi.edu.tr (C.K.A.)

**Keywords:** diabetes mellitus, immediate dentin sealing, shear bond strength, resin cementation

## Abstract

This in vitro study evaluated the shear bond strength (SBS) of resin cement to diabetic and non-diabetic dentin following immediate dentin sealing (IDS) or conventional bonding without IDS, under two cementation protocols: immediate cementation (IC) and one-week delayed cementation (DC) after provisionalization. From a biomimetic perspective, IDS represents a tissue-preserving adhesive approach aimed at maintaining a stable resin–dentin interface; however, systemic conditions such as diabetes mellitus may alter the dentin substrate and affect adhesive performance. A total of 120 extracted human molars were divided into diabetic (*n* = 60) and non-diabetic (*n* = 60) groups, each further categorized by adhesive strategy (IDS or without IDS) and cementation protocol (IC or DC). IDS was performed using a three-step etch-and-rinse adhesive system, and cementation was completed with a dual-cure resin cement. Differences in SBS were observed between diabetic and non-diabetic dentin, adhesive strategies, and cementation protocols. Across all conditions, IDS-treated specimens exhibited higher bond strength than those without IDS, and DC resulted in lower SBS than IC in both adhesive strategies. In all subgroups, non-diabetic dentin showed greater SBS than diabetic dentin. The highest SBS was recorded in the non-diabetic IDS-IC group, whereas the lowest was observed in the diabetic without IDS–DC group. Under the tested laboratory conditions, IDS-treated groups showed higher SBS than the corresponding groups without IDS, while the immediate cementation protocol resulted in higher SBS than the delayed cementation protocol.

## 1. Introduction

Diabetes mellitus (DM) has been shown to induce detrimental biochemical and structural modifications within oral tissues, including the dentin substrate, characterized by enlarged dentinal tubules, disrupted peritubular calcification, and the accumulation of advanced glycation end-products (AGEs) within the collagen matrix [1,2,3]. Additionally, diabetes has been shown to increase the expression of matrix metalloproteinases (MMPs) [4]; such elevated MMP activity may promote degradation of the dentin collagen matrix and ultimately compromise bond durability. Given these alterations, resin–dentin bond strength in diabetic dentin may be adversely affected; however, the influence of adhesive protocols under diabetic conditions remains inadequately clarified.

The longevity of indirect restorations depends on the stability and durability of the resin–dentin–adhesive interface [5]. Owing to its structural heterogeneity, high organic content, and fluid-filled tubules, dentin is a dynamic substrate that is inherently less favorable for adhesion than enamel and can impair optimal resin infiltration [6,7,8]. A stable interface is nonetheless essential for restoration retention and for minimizing microleakage, postoperative sensitivity, and secondary caries [9].

In conventional indirect procedures, dentin bonding is performed at definitive cementation, after a provisionalization period during which the dentin is exposed to saliva, temporary cements, and biofilm contaminants that can compromise the adhesive interface [10,11,12]. The Immediate Dentin Sealing (IDS) strategy addresses this by sealing freshly prepared dentin with an adhesive immediately after preparation, before provisionalization or impression [13], allowing a well-polymerized hybrid layer to form under optimal, contamination-free conditions [11]. IDS has been shown to improve resin–dentin bond strength, marginal adaptation, and postoperative sensitivity relative to the conventional approach [14,15,16,17].

Among the adhesive systems used for IDS [13,14], the filler-containing three-step etch-and-rinse adhesive OptiBond FL (Kerr) is preferred for its uniform layer, cohesiveness with the luting resin, and resistance to air abrasion [18,19], whereas simpler self-etch adhesives form thinner, mechanically weaker layers more prone to removal and dentin re-exposure during surface cleaning [20].

When treatment requires more than one visit, effective removal of temporary cement is critical for bonding [15]. Various cleansing protocols, including pumice polishing, air polishing, air abrasion, and manual or ultrasonic instruments, have been proposed for this purpose [21,22]. Air abrasion with aluminum oxide (Al_2_O_3_) particles, in particular, can remove surface contaminants and increase surface area, thereby enhancing wettability and helping to reestablish a clean and receptive bonding interface [23,24].

Biomimetic restorative approaches aim to preserve dental tissues and maintain the structural and functional integrity of the tooth–restoration complex. Within this framework, IDS may be regarded as a biomimetically oriented adhesive strategy because it seals freshly prepared dentin immediately after preparation, before contamination and provisionalization, thereby promoting the establishment of a stable resin–dentin interface [13,14,18]. By maintaining a protected dentin substrate and reducing the potential for surface alteration during subsequent restorative procedures, IDS is consistent with the tissue-preserving principles of biomimetic restorative dentistry [14,19].

Although adhesive strategies have been extensively studied in healthy dentin, limited evidence is available regarding their performance in dentin affected by systemic conditions such as diabetes mellitus. In particular, the effects of immediate dentin sealing and cementation protocol on bonding to diabetic dentin have not been investigated. Therefore, this in vitro study aimed to determine the shear bond strength (SBS) of resin cement to dentin surfaces obtained from diabetic (D) and non-diabetic (nD) teeth, following IDS or without IDS protocols, under two simulated clinical conditions: immediate cementation (IC) and one-week delayed cementation (DC). The novelty of the study lies in the combined evaluation of diabetic dentin, the IDS versus without IDS adhesive strategy, and the immediate versus delayed cementation protocol, enabling the interactions among these factors to be assessed specifically under diabetic conditions. The findings are expected to provide clinically relevant insights for improving adhesive protocols in patients with DM and enhancing the long-term performance of indirect restorations.

The study was conducted based on the following three null hypotheses:

**H_1_.**  *The SBS of resin cement does not significantly differ between diabetic dentin and non-diabetic dentin.*

**H_2_.**  *IDS has no significant effect on the SBS of resin cement to dentin.*

**H_3_.**  *Cementation protocol (IC vs. DC) has no significant effect on SBS of resin cement to dentin.*

## 2. Materials and Methods

**Figure 1:**
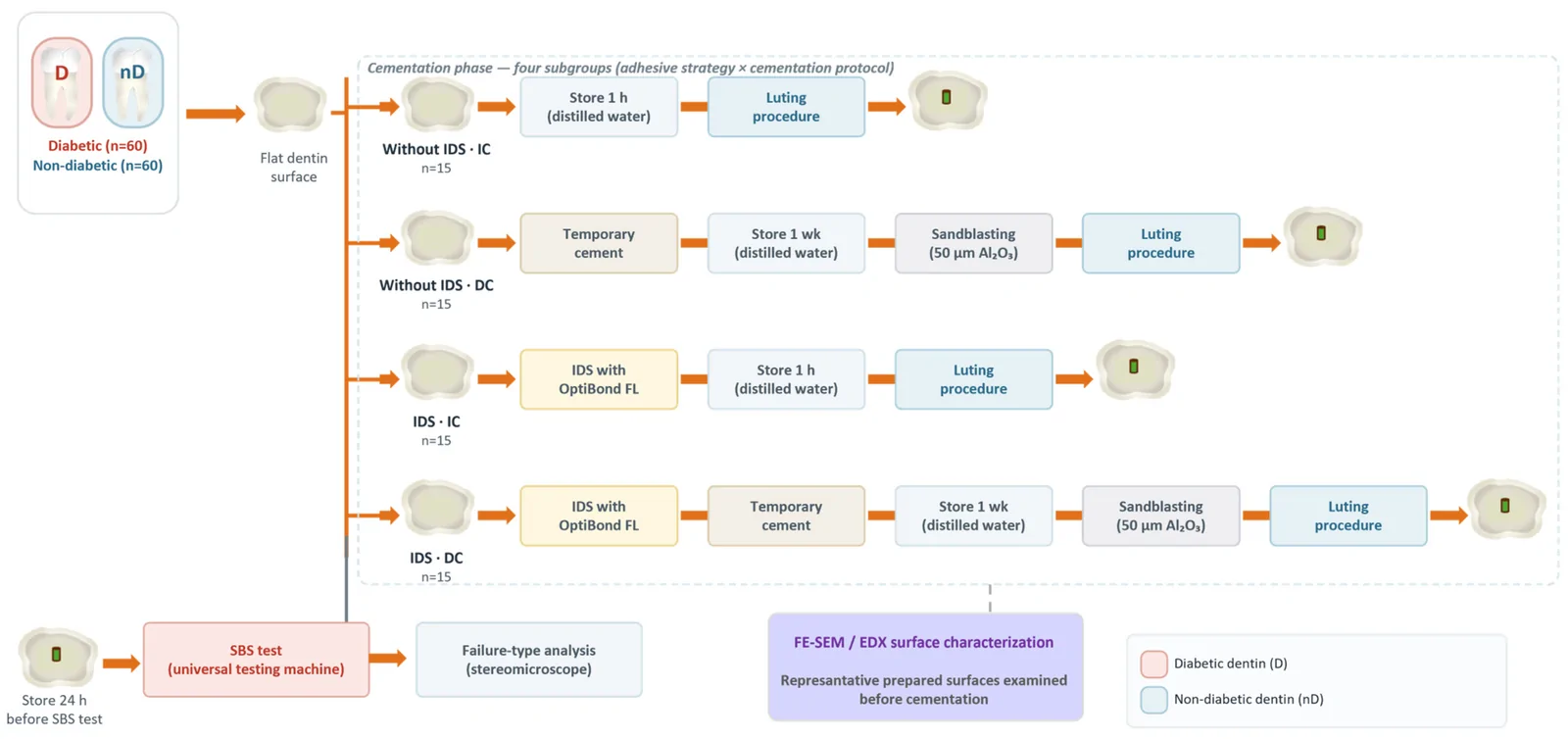
Schematic illustration of the experimental design and workflow.

The materials used in the study and their compositions are listed in Table 1, while the experimental workflow is illustrated in Figure 1.

### 2.1. Tooth Selection and Specimen Preparation

Ethical approval for this in vitro study was obtained from the institutional ethics committee. A power analysis was performed using G*Power software (version 3.1.9.7; Franz Faul, University of Kiel, Germany). The sample size was determined a priori for the main effects and interactions of the three-way ANOVA design. Assuming a medium effect size of f = 0.30, a significance level of α = 0.05, and a target power of 0.90, the analysis indicated a required total sample size of 120 specimens (15 per subgroup).

The study was carried out using 120 freshly extracted human molars, comprising 60 teeth obtained from patients with a documented diagnosis of diabetes mellitus (diabetic; D) and 60 from systemically healthy individuals (non-diabetic; nD). Only permanent molars extracted from adult donors (aged 25–65 years) were included; all teeth in the diabetic group were obtained from patients with a physician-confirmed diagnosis of diabetes mellitus. Each of the 120 teeth was obtained from a separate donor (one tooth per individual). All teeth were obtained as anonymized biological waste material from extractions performed for clinical reasons unrelated to this study, including periodontal or restorative indications; no patient-identifiable information was collected. Teeth were assigned to the diabetic or non-diabetic group based on the patients’ medical records; a tooth was classified as diabetic only when a physician-confirmed diagnosis of diabetes mellitus was present in the record at the time of extraction. Teeth were included if they were caries-free, unrestored, and structurally intact. Teeth with caries, restorations, cracks, structural defects, sclerotic dentin, or previous endodontic treatment were excluded. Soft tissue residues were removed, and the teeth were cleaned thoroughly. After extraction, the teeth were disinfected and stored in 0.1% thymol solution at 4 °C [25] and used within three months of extraction. The nD (*n* = 60) and D (*n* = 60) specimens were each divided into two groups according to the application of immediate dentin sealing (IDS) or without IDS (*n* = 30). Each group was then further subdivided according to cementation protocol into immediate cementation (IC; after 1 h) and delayed cementation (DC; after 1 week) subgroups (*n* = 15). Allocation of specimens to the dentin-sealing (IDS vs. without IDS) and cementation (IC vs. DC) subgroups was performed by simple randomization using a computer-generated random sequence, with equal numbers assigned to each subgroup [26].

The occlusal enamel of each tooth was removed to obtain flat mid-coronal dentin using a water-cooled low-speed diamond disc. The dentin surfaces were then polished sequentially with 600, 800, and 1000-grit silicon carbide abrasive papers using a polishing machine (PRESI, Mecapol, P230, Grenoble, France) to ensure a uniform smear layer and to standardize the dentin surface [27]. Each specimen was embedded in self-cured acrylic resin blocks, ensuring that the dentin surfaces remained exposed.

### 2.2. IDS Procedure

The IDS procedure was performed on freshly prepared dentin surfaces using a standardized protocol with OptiBond FL (Kerr; USA), as described in previous studies [18,28]. The detailed application steps are presented in Table 2.

### 2.3. Cementation Procedures

Cementation procedures were performed under two different protocols: immediate cementation (IC) and delayed cementation (DC). The detailed application steps are presented in Table 3.

### 2.4. SBS Test and Failure Analysis

Following cementation, all samples were stored in distilled water at 37 °C for 24 h. Shear bond strength (SBS) testing was conducted using a universal testing machine (Shimadzu AG-IS Autograph, Shimadzu Scientific Instruments, Kyoto, Japan) at a crosshead speed of 1 mm/min. The force at failure (in Newtons) was recorded and converted to megapascals (MPa) by dividing by the bonded area (1.131 mm^2^). The failure modes were examined under a microscope at 25× magnification and classified as follows: adhesive failure at the dentin–adhesive interface (Type 1), adhesive failure at the adhesive–resin cement interface (Type 2), and mixed failure (Type 3). The operator performing the shear bond strength testing and the examiner classifying the failure modes were blinded to group allocation, with specimens coded by an independent investigator.

### 2.5. Field-Emission Scanning Electron Microscopy (FE-SEM) Analysis

FE-SEM analysis was performed to assess the surface morphology of selected diabetic and non-diabetic dentin specimens after IDS application, provisionalization, and airborne-particle abrasion procedures. Representative specimens were selected from IDS-treated surfaces, IDS-treated surfaces after 1-week provisionalization with temporary cement followed by airborne-particle abrasion, and non-IDS surfaces after 1-week provisionalization with temporary cement followed by airborne-particle abrasion. The samples were affixed to standard SEM stubs using carbon conductive adhesive tape. To enhance surface conductivity, the samples were coated with approximately 2 nm of AuPd alloy by diffusion sputtering, using a Leica EM ACE 200 (Leica Microsystems CMS GmbH, Wetzlar, Germany) system under high vacuum conditions. High-resolution imaging was conducted using a Carl Zeiss Sigma 300 VP (Carl Zeiss Microscopy GmbH, Oberkochen, Germany) field-emission scanning electron microscope (FE-SEM) operated under high vacuum conditions. Imaging parameters included an accelerating voltage of 20.01 kV, a working distance ranging from 12 to 15 mm, and a diaphragm aperture size of 30 μm. A secondary electron detector referenced with a Polaroid 545 system was employed. Each image, acquired at a resolution of 1024 × 768 pixels, had a scan duration of 5.4 s. Three specimens per group were randomly selected for FE-SEM analysis. For each specimen, at least three fields were examined and imaged at a standardized magnification of 2000×. Image acquisition and interpretation were performed by an examiner blinded to group allocation.

### 2.6. Statistical Analysis

Statistical analysis of the data was performed using SPSS software (IBM SPSS Statistics, Version 26). The assumption of normality was evaluated using the Kolmogorov–Smirnov and Shapiro–Wilk tests, which confirmed that the data were normally distributed. Homogeneity of variance was assessed using Levene’s test, which indicated that the assumption of equal variances was satisfied (*p* = 0.085). A three-way analysis of variance (ANOVA) was used to evaluate the combined effects of dentin type (diabetic vs. non-diabetic), adhesive strategy (IDS vs. without IDS), and cementation protocol (IC vs. DC) on shear bond strength. Given the significant three-way interaction, simple-effects pairwise comparisons were performed using estimated marginal means with Bonferroni adjustment for multiple comparisons. Differences were regarded as statistically significant when *p* < 0.05.

## 3. Results

**Figure 2:**
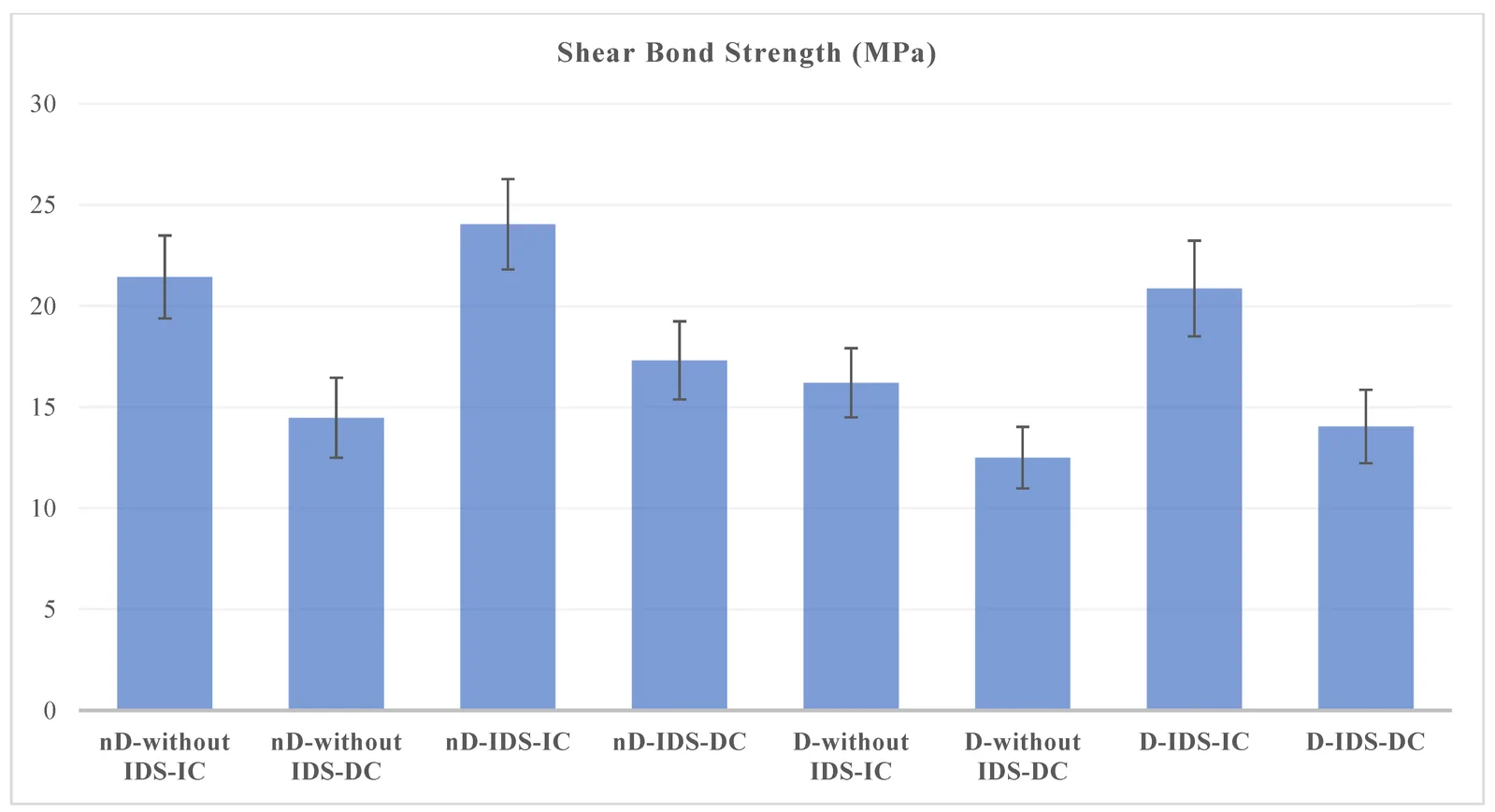
Shear bond strength values for diabetic and non-diabetic dentin according to adhesive strategy and cementation protocol. Bars represent mean ± SD.

Statistically significant differences in bond strength were observed among different dentin conditions (D vs. nD), adhesive strategies (IDS vs. without IDS), and cementation protocols (IC vs. DC) (*p* < 0.05) (Table 4) (Figure 2).

Across all adhesive strategy and cementation protocol combinations, non-diabetic dentin exhibited higher SBS values than diabetic dentin (*p* < 0.05). For both diabetic and non-diabetic dentin, IDS resulted in the highest bond strength under each cementation protocol, although the magnitude of this effect varied with dentin condition and cementation protocol, consistent with the significant interactions (*p* < 0.05). Furthermore, IC produced higher bond strength than DC under both adhesive strategies, though the size of this difference varied across conditions (*p* < 0.05).

No significant interaction was found between dentin type and adhesive strategy (*p* = 0.600), indicating that the effect of IDS was similar in both diabetic and non-diabetic dentin (Table 4). However, significant interactions were observed between dentin type and cementation protocol (*p* = 0.030), as well as between adhesive strategy and cementation protocol (*p* = 0.045). Furthermore, the three-way interaction among dentin type, adhesive strategy, and cementation protocol was statistically significant (*p* = 0.022), indicating that the effect of IDS on bond strength was influenced by both dentin condition and cementation protocol (Table 4). Because this three-way interaction was significant, the main effects of IDS and of the cementation protocol should be interpreted in the light of this interaction rather than as uniform effects acting equally across all conditions; the direction and magnitude of each effect therefore depended on the specific combination of dentin condition and cementation protocol.

Because the three-way interaction was significant, subgroup differences were examined as simple-effects pairwise comparisons derived from the three-way ANOVA model, using estimated marginal means with Bonferroni adjustment for multiple comparisons (Table 5). Non-diabetic dentin showed significantly higher SBS than diabetic dentin in every adhesive–cementation combination, but the magnitude of this difference varied with condition, ranging from 1.97 MPa in the without IDS–DC subgroup (95% CI 0.541–3.397; adjusted *p* = 0.007) to 5.24 MPa in the without IDS–IC subgroup (95% CI 3.809–6.665; adjusted *p* < 0.001).

IDS produced significantly higher SBS than the corresponding without IDS subgroup in all four dentin–cementation combinations, although the effect size again differed by condition; it was smallest in diabetic–DC dentin (1.54 MPa; 95% CI 0.107–2.963; adjusted *p* = 0.035) and largest in diabetic–IC dentin (4.67 MPa; 95% CI 3.238–6.094; adjusted *p* < 0.001). Likewise, IC yielded significantly higher SBS than DC in every dentin–adhesive combination (all adjusted *p* < 0.001), with mean differences ranging from 3.70 MPa in diabetic without IDS dentin (95% CI 2.269–5.125) to 6.97 MPa in non-diabetic without IDS dentin (95% CI 5.537–8.393). Consistent with the significant interactions, the highest mean SBS occurred in the non-diabetic IDS–IC subgroup (24.03 ± 2.23 MPa) and the lowest in the diabetic without IDS–DC subgroup (12.49 ± 1.52 MPa).

**Figure 3:**
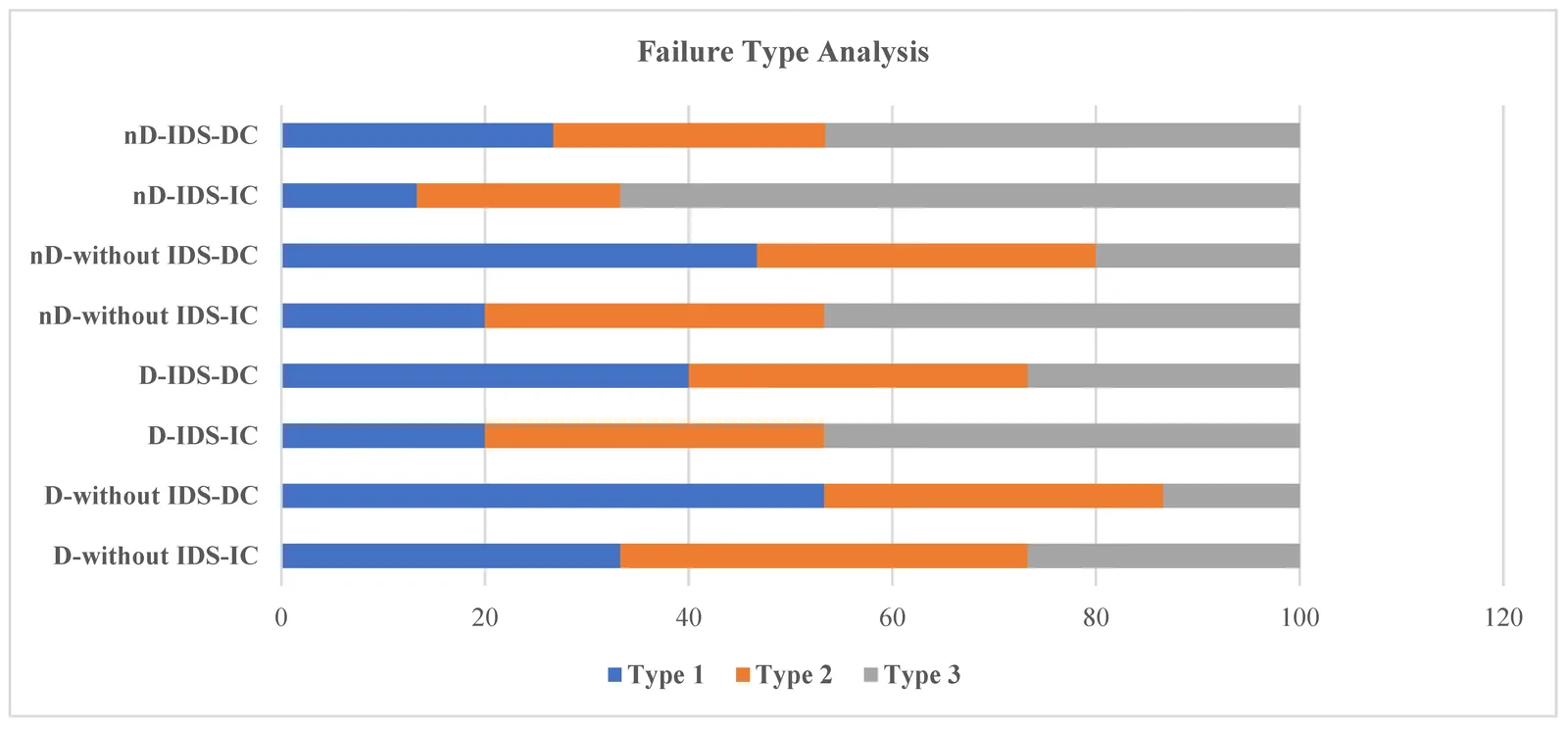
Failure Type Analysis (Type 1: adhesive failure at dentin–adhesive interface, Type 2: adhesive failure at adhesive–resin cement interface, Type 3: mixed).

Failure types, classified as adhesive failures at the dentin–adhesive interface (Type 1), adhesive failures at the adhesive–resin cement interface (Type 2), and mixed failure (Type 3), are shown in Figure 3. Failure-mode distributions were analyzed descriptively. Therefore, the percentages reported below reflect observed tendencies rather than statistically significant differences. The highest percentages of mixed failures (Type 3) were observed in the nD-IDS-IC (66.7%), nD–without IDS-IC (46.7%), D-IDS-IC (46.7%), and nD-IDS–DC (46.6%) groups. Adhesive failures at the dentin–adhesive interface (Type 1) were most frequent in the D–without IDS–DC (53.3%), nD–without IDS-IC (46.7%), and D-IDS-IC (40.0%) groups, whereas adhesive failures at the adhesive–resin cement interface (Type 2) were most frequently observed in the D–without IDS-IC (40.0%) group.

## 4. Discussion

The primary objective of this in vitro study was to evaluate the effect of different adhesive strategies (IDS vs. without IDS) and cementation protocols (IC vs. DC) on the SBS of resin cement to diabetic and non-diabetic dentin. Based on the obtained results, diabetic dentin groups resulted in lower bond strength, delayed cementation groups exhibited lower bonding performance than immediate cementation groups, and the application of IDS resulted in higher bond strength than the groups without IDS. The highest bond strength was observed in the non-diabetic IDS–IC group, whereas the lowest bond strength was recorded in the diabetic without IDS–DC group.

One of the most noteworthy findings of this study is that diabetic dentin groups consistently exhibited lower bond strengths than their non-diabetic counterparts, regardless of the adhesive strategy or cementation protocol used. This result is consistent with previous literature reporting structural and biochemical alterations in dentin tissue associated with DM, a systemic condition characterized by chronic hyperglycemia [2,3]. According to the literature, DM has been associated with enlargement of dentinal tubules [2], disruption of peritubular calcification [1,31], and accumulation of AGEs within the collagen matrix [3]. The accumulation of AGEs in dentin has also been reported to correlate with reduced flexural strength and mechanical toughness in both coronal and radicular regions of the tooth [32,33], and increased MMP activity has been proposed as a further contributor to collagen degradation that may compromise the durability of the resin–dentin interface [4]. It should be emphasized, however, that tubular diameter and density, AGE content, collagen characteristics, MMP activity, microhardness, and chemical composition were not directly measured in the specimens used in the present study; these mechanisms are therefore offered as plausible explanations drawn from previous literature rather than as effects experimentally demonstrated here.

Our findings suggest that dentin in diabetic patients may represent a less favorable substrate for adhesive procedures, and the first null hypothesis was therefore rejected. Previous studies comparing the bond strength of deep and superficial dentin have consistently demonstrated inferior adhesion in deep dentin [34,35]. In a recent study, endodontic sealers were reported to penetrate diffusely into the enlarged dentinal tubules observed in diabetic dentin, and residual sealer remnants that could not be effectively removed were found to adversely affect the bonding of dentin to adhesive resins [36]. In this context, the decreased bond strength observed in diabetic dentin in the present study may be partially explained by morphological similarities between diabetic and deep dentin reported in the literature, although it is likely a multifactorial phenomenon that our data cannot resolve directly. However, IDS was associated with higher SBS in diabetic dentin under the tested conditions, suggesting that this adhesive strategy may improve bonding performance in this substrate.

The IDS technique has been reported to enhance bond strength and promote the formation of a more stable hybrid layer by applying the adhesive immediately onto freshly cut dentin [37,38,39]. The infiltration of adhesive agents into freshly prepared dentin prevents the collapse of collagen fibers and effectively seals the dentinal tubules, thereby providing protection against contamination from saliva, microorganisms, or provisional cement [13,16,40]. These favorable conditions contribute to the development of a uniform and durable hybrid layer, which enhances micromechanical retention. However, certain studies have questioned the superiority of IDS, suggesting that it may not consistently offer significant advantages over the conventional approach (without IDS) [24,41]. Carvalho et al. [19] reported that the bond strength improvement provided by IDS was observed only when a three-step etch-and-rinse adhesive was used, with the filled OptiBond FL showing superior performance over unfilled or lightly filled systems, while other adhesive types did not produce significant differences between IDS protocols. Filho et al. [42] compared multiple adhesive systems applied with or without the IDS procedure; the results showed that the initial bond strength advantages of IDS observed at 7 days of water storage were no longer statistically significant after a 3-month aging period. These findings suggest that the long-term advantages of IDS may be material-dependent and may diminish over time.

**Figure 4:**
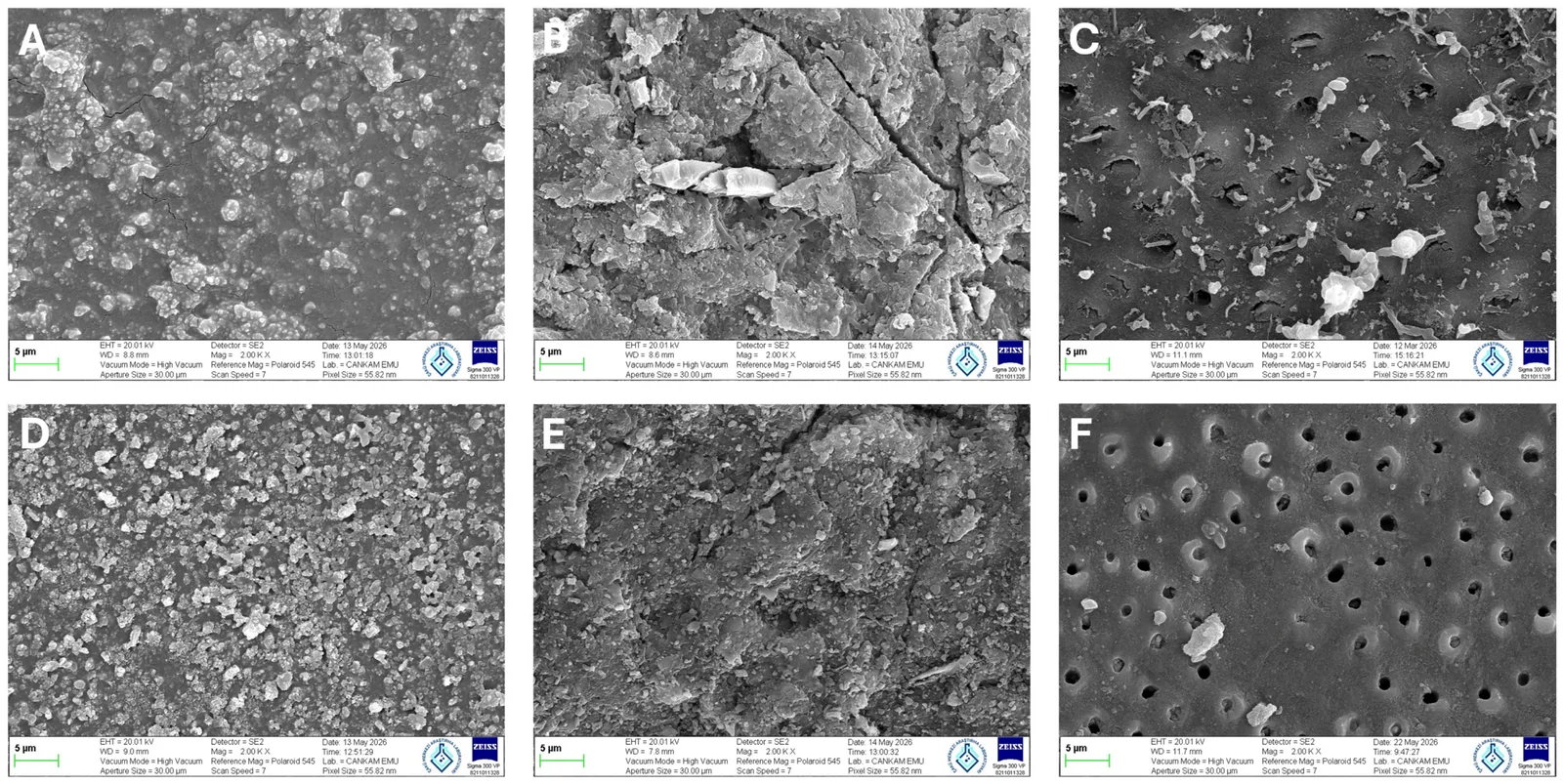
Representative FE-SEM images of diabetic and non-diabetic dentin surfaces following different adhesive and provisionalization procedures. (**A**,**D**): Immediate dentin sealing (IDS) surfaces. (**B**,**E**): IDS-treated dentin surfaces after 1-week provisionalization and airborne-particle abrasion. (**C**,**F**): Dentin surfaces without IDS after 1-week provisionalization and airborne-particle abrasion. (**A**–**C**): Diabetic dentin. (**D**–**F**): Non-diabetic dentin.

In three-step etch-and-rinse adhesive systems, the OptiBond FL adhesive is considered the gold standard due to its superior bonding performance and long-term reliability [43,44,45]. This favorable performance has been linked to the high filler content and the presence of GPDM in OptiBond FL, which chemically interacts with hydroxyapatite and promotes the formation of a mechanically robust and durable interface between the hybrid layer and the overlying restoration. In the present study, OptiBond FL was used during the application of the IDS protocol. The results demonstrated that IDS significantly increased the bond strength in both diabetic and non-diabetic dentin groups. Similarly, within both the immediate (IC) and delayed (DC) cementation groups, the application of IDS resulted in improved bonding performance. Therefore, the second null hypothesis, which stated that the adhesive strategy (IDS vs. without IDS) does not significantly influence the SBS of resin cement to dentin, was rejected. Representative FE-SEM observations were consistent with the SBS findings, as IDS-treated dentin surfaces showed greater surface coverage and reduced visibility of dentinal tubule openings in both diabetic and non-diabetic specimens (Figure 4A,D). These qualitative observations should be interpreted as descriptive rather than confirmatory evidence. This morphological observation is consistent with the higher SBS values observed in IDS-treated groups and supports the role of IDS in preserving a more favorable bonding substrate.

In the literature, both microtensile and shear bond strength tests have been widely employed to evaluate the effect of immediate dentin sealing (IDS) on bond strength [14,46]. While microtensile testing is often preferred due to its higher sensitivity, shear bond strength (SBS) testing remains a well-established and clinically relevant method for evaluating adhesive performance. In the present study, SBS testing was employed, and the SBS values obtained in the IDS-applied groups were comparable to those reported in previous studies utilizing similar testing methodologies [30,47,48]. This consistency with the existing literature supports the validity of the present findings and further confirms the positive contribution of IDS to bond strength under the tested conditions. Furthermore, in diabetic dentin groups, the application of IDS significantly increased bond strength in both immediate and delayed cementation groups. Therefore, IDS in diabetic dentin appears to be a promising strategy for reducing the detrimental effects of diabetes-related structural and biochemical alterations on resin–dentin bonding.

The cementation protocol and the effective removal of provisional cement prior to definitive bonding play a critical role in ensuring optimal adhesive performance. Magne et al. [45] demonstrated that in specimens treated with IDS using OptiBond FL, clinically acceptable bond strength values were preserved even when definitive cementation was delayed for up to 12 weeks, as long as the provisional cement was removed using APA with aluminum oxide. APA with aluminum oxide is known to increase surface energy and roughness, which can facilitate better resin penetration and hybrid layer formation [49,50]. However, recent evidence suggests that the mechanical impact of APA may be detrimental to the integrity of the IDS layer. Systematic reviews report that aggressive air-abrasion protocols may thin or even completely remove the polymerized adhesive layer, particularly when it is initially thin, thereby disrupting the sealed dentin interface and leading to a significant reduction in bond strength [14,51]. Furthermore, high-pressure APA applications have been shown to induce microcrack formation within the IDS layer and weaken its surface structure [52]. In a previous study [23], various techniques for removing temporary cement were evaluated in specimens that had undergone IDS. The findings indicated that manual removal using instruments such as curettes was insufficient to fully eliminate residual temporary cement from the IDS surface. However, when this procedure was combined with additional surface treatments such as APA with aluminum oxide or the use of a diamond bur, more effective decontamination was achieved. Moreover, these surface treatments were found to enhance the wettability of the adhesive surface and create micro-irregularities that increased the available bonding area, ultimately contributing to improved bond strength.

It is important to emphasize that, in this study, the immediate (IC) and delayed (DC) groups differed not only in the elapsed time before definitive cementation but also in a series of procedures applied only to the DC groups, namely, provisional cement application, one week of storage, provisional cement removal, and APA with aluminum oxide. The DC condition therefore represents a composite cementation/provisionalization protocol rather than an isolated effect of time, and the differences described below should be interpreted as the combined effect of these procedural steps rather than of elapsed time alone. In the present study, provisional cement was removed using an excavator followed by APA with aluminum oxide, and lower bond strength was observed in all DC groups compared with their IC groups. Therefore, the third null hypothesis, which stated that the cementation protocol does not significantly affect the SBS of resin cement to dentin, was rejected. Regardless of dentin condition (diabetic or non-diabetic) and adhesive strategy (with or without IDS), the immediate cementation protocol was associated with significantly higher bond strength than the delayed cementation protocol. Notably, the lowest bond strength was observed in the diabetic without IDS–DC group, whereas immediate cementation of the same group resulted in a marked increase in bond strength. This outcome may reflect the incomplete and ineffective removal of provisional cement from the bonding surface, together with the potential adverse effects of residual aluminum oxide particles remaining on the surface after APA, rather than the passage of time as such. Similarly, within the IDS groups, specimens subjected to the delayed cementation protocol exhibited lower bond strength than those subjected to the immediate cementation protocol. This difference may likewise be attributed to the mechanical trauma caused by APA during provisional cement removal, which may have partially stripped the thin IDS layer or induced microcracks, thereby weakening the structural integrity of the adhesive interface. Because these procedural components were inherent to the delayed cementation protocol and were not varied independently, the present design cannot isolate the contribution of any single step, and the reduced SBS in the DC groups should be understood as a composite effect.

Representative FE-SEM observations of IDS-treated surfaces after 1-week provisionalization and subsequent airborne-particle abrasion showed increased surface irregularities and deposits consistent with residual temporary cement or debris generated during the abrasion procedure (Figure 4B,E). In the non-IDS groups subjected to provisionalization and airborne-particle abrasion, dentin surfaces also exhibited irregular morphology and surface deposits (Figure 4C,F). These qualitative findings were consistent with the lower SBS values observed in the delayed cementation groups; however, no causal relationship can be established because quantitative SEM analysis was not performed. Therefore, the FE-SEM findings should be interpreted as descriptive morphological observations rather than as direct evidence of the mechanisms responsible for the observed differences in bond strength.

Certain limitations exist within this laboratory-based research that warrant caution when analyzing the findings. Primarily, the experimental setup could not flawlessly simulate the dynamic conditions of the intraoral environment, including pulpal pressure, masticatory forces, thermal fluctuations, salivary enzymes, and pH changes. Additionally, impression-taking procedures prior to temporization were not simulated, and only one adhesive system (OptiBond FL) and one resin cement (RelyX Ultimate) were used, which limits the generalizability of the findings to other materials. The aging protocol was limited to one week of distilled water storage, without thermocycling or cyclic loading. Furthermore, although a subset of the teeth used in this study was obtained from patients with a confirmed diagnosis of diabetes mellitus, diabetic characterization data (e.g., diabetes type and duration, HbA1c, medication) were not available, which should be considered when interpreting the results. A further limitation is inherent to the study design: because the delayed-cementation (DC) groups differed from the immediate-cementation (IC) groups not only in elapsed time but also in provisional cement application, one week of storage, provisional cement removal, and airborne-particle abrasion, the effect of cementation protocol cannot be isolated from these accompanying provisionalization and surface-cleaning procedures; the reduced bond strength observed in the DC groups should therefore be interpreted as a composite effect of the delayed protocol rather than of time alone. Finally, the FE-SEM evaluation was qualitative and descriptive; surface features were not quantified, and the micrographs illustrate representative surface morphologies.

## 5. Conclusions

This in vitro study demonstrated that dentin condition, adhesive strategy, and cementation protocol were each significantly associated with differences in resin–dentin bond strength, with the magnitude of these effects varying across conditions, consistent with the significant three-way interaction. Bond strength was consistently lower in diabetic dentin. Under the tested laboratory conditions, IDS resulted in higher bond strength than the corresponding conditions without IDS in both diabetic and non-diabetic dentin. Immediate cementation, performed one hour later, also provided higher bond strength than the delayed cementation protocol. Collectively, under the tested laboratory conditions, combining immediate dentin sealing with immediate cementation yielded the highest resin–dentin bond strength in diabetic dentin. These preliminary in vitro findings warrant further aging studies and clinical investigations before clinical recommendations can be established.

## Figures and Tables

**Table 1:** The materials, manufacturers and composition used in the study.

Material	Manufacturer (Lot Number)	Composition
Scotchbond Universal Etchant	3M Deutschland GmbH, Neuss, Germany (9985311)	32 wt% phosphoric acid, 60% water, 5% synthetic amorphous silica
OptiBond FL	Kerr, Orange, CA, USA (9558965)	Primer: HEMA, GPDM, MMEP, ethanol, water, initiators Adhesive: Bis-GMA, HEMA, GPDM, barium-aluminum, borosilicate glass, disodium hexaflurosilicate, fumed silica (48% filler)
Single Bond Universal Adhesive	3M Deutschland GmbH, Neuss, Germany (30510D)	10-MDP phosphate monomer, Vitrebond, copolymer, HEMA, Bis-GMA, dimethacrylate resin, camphorquinone, silane, ethanol, and water
RelyX Ultimate Clicker Resin Cement	3M Deutschland GmbH, Neuss, Germany (9909759)	Base: methacrylate monomers, radiopaque silanated fillers, initiators, stabilizers, rheologic additives Catalyst: methacrylate monomers, radiopaque alkaline fillers, initiators, stabilizers, pigments, rheologic additives, fluorescent dye, activator
Temp-Bond NE (Kerr; USA)	Kerr, Orange, CA, USA (8485854)	Base paste: zinc oxide, mineral oil, lecithin, corn starch and iron oxide pigments. Catalytic paste: polyorganic acids.
DENTO-PREP Sandblaster	Ronvig Dental, Denmark	Al_2_O_3_ particles; particle size 50 μm

Abbreviations: HEMA (hydroxyethyl methacrylate), GPDM (glycerol phosphate dimethacrylate), MMEP (methacryloyloxyethyl phosphate), Bis-GMA (bisphenol A–glycidyl methacrylate), 10-MDP (10-methacryloyloxydecyl dihydrogen phosphate).

**Table 2:** Immediate dentin sealing (IDS) protocol.

Step	Procedure
Acid etching	37% phosphoric acid applied for 15 s, rinsed with water for 20 s, and gently air-dried for 3 s (without desiccation).
Primer application	OptiBond FL primer actively applied for 15 s, followed by gentle air-drying for 5 s to allow solvent evaporation.
Adhesive application	OptiBond FL adhesive applied for 15 s without air-thinning. Light-cured for 20 s using an LED curing unit (D-Light Pro, GC, Tokyo, Japan); oxygen inhibition was removed by applying a glycerin barrier followed by additional light activation for 10 s and water rinsing [19].

**Table 3:** Cementation protocols used in the study.

Procedure	Immediate Cementation (IC)	Delayed Cementation (DC)
Storage before cementation	Specimens stored in distilled water for 1 h [29].	Surfaces were covered with Temp-Bond NE temporary cement applied at a standardized thickness of 2.0 mm. A constant load of 500 g was applied for 60 s using a glass slab [23]. The specimens were then stored in distilled water at 37 °C for 1 week [29].
Temporary cement removal	Not applicable.	Temp-Bond NE temporary cement removed using an excavator followed by airborne-particle abrasion with 50 µm Al_2_O_3_ for 5 s at 1 cm distance under 2 bar pressure [19]. Following air abrasion, the surfaces were thoroughly rinsed with an air–water spray and were subsequently gently air-dried.
Luting procedure	Single Bond Universal adhesive applied for 20 s and gently air-thinned for 5 s (no light curing); RelyX Ultimate resin cement placed into tygon tubes and light-cured for 20 s [30].	Same procedure as IC.

**Table 4:** Three-way analysis of variance (ANOVA) for the effects of dentin condition, adhesive strategy, and cementation protocol on shear bond strength (SBS).

Source of Variation	df	Mean Square	F	*p*	Partial η^2^
**Main effects**					
Dentin condition	1	349.491	89.715	<0.001	0.445
Adhesive strategy	1	254.189	65.251	<0.001	0.368
Cementation protocol	1	1100.557	282.516	<0.001	0.716
**Two-way interactions**					
Dentin × Adhesive	1	1.077	0.277	0.600	0.002
Dentin × Cementation	1	18.937	4.861	0.030	0.042
Adhesive × Cementation	1	15.820	4.061	0.046	0.035
**Three-way interaction**					
Dentin × Adhesive × Cementation	1	21.143	5.427	0.022	0.046
Error	112	3.896			
Total	119				

Type III sum of squares. Statistically significant effects (*p* < 0.05) are indicated by *p*-values in the table; the model explained 80.1% of the total variance (R^2^ = 0.801, adjusted R^2^ = 0.789). df, degrees of freedom; η^2^, eta squared. Cementation protocol refers to immediate (IC) versus delayed (DC) cementation.

**Table 5:** Bonferroni-adjusted simple-effects comparisons of shear bond strength (SBS) according to dentin condition, adhesive strategy, and cementation protocol.

Simple-Effect Comparison	Group 1, Mean ± SD (MPa)	Group 2, Mean ± SD (MPa)	Mean Difference (MPa)	95% CI of Difference	Adjusted *p*
**Dentin condition**					
nD vs. D, without IDS–DC	14.46 ± 1.98	12.49 ± 1.52	1.969	0.541 to 3.397	0.007
nD vs. D, without IDS–IC	21.43 ± 2.05	16.19 ± 1.71	5.237	3.809 to 6.665	<0.001
nD vs. D, IDS–DC	17.30 ± 1.93	14.03 ± 1.82	3.269	1.841 to 4.697	<0.001
nD vs. D, IDS–IC	24.04 ± 2.23	20.86 ± 2.37	3.179	1.751 to 4.607	<0.001
**Adhesive strategy**					
IDS vs. without IDS, nD–DC	17.30 ± 1.93	14.46 ± 1.98	2.835	1.407 to 4.263	<0.001
IDS vs. without IDS, nD–IC	24.04 ± 2.23	21.43 ± 2.05	2.608	1.180 to 4.036	<0.001
IDS vs. without IDS, D–DC	14.03 ± 1.82	12.49 ± 1.52	1.535	0.107 to 2.963	0.035
IDS vs. without IDS, D–IC	20.86 ± 2.37	16.19 ± 1.71	4.666	3.238 to 6.094	<0.001
**Cementation protocol**					
IC vs. DC, nD–without IDS	21.43 ± 2.05	14.46 ± 1.98	6.965	5.537 to 8.393	<0.001
IC vs. DC, nD–IDS	24.04 ± 2.23	17.30 ± 1.93	6.738	5.310 to 8.166	<0.001
IC vs. DC, D–without IDS	16.19 ± 1.71	12.49 ± 1.52	3.697	2.269 to 5.125	<0.001
IC vs. DC, D–IDS	20.86 ± 2.37	14.03 ± 1.82	6.828	5.400 to 8.256	<0.001

Values are presented as mean ± SD. Simple-effects pairwise comparisons were derived from the three-way ANOVA model using estimated marginal means with Bonferroni adjustment for multiple comparisons. Mean differences are presented with 95% confidence intervals. nD: non-diabetic dentin; D: diabetic dentin; IDS: immediate dentin sealing; IC: immediate cementation protocol; DC: delayed cementation protocol.

## Data Availability

The article itself contains the information needed to support its conclusions. Upon reasonable request, the corresponding author will make the dataset available.
